# Serendipitous Supernormality

**DOI:** 10.1111/anec.70069

**Published:** 2025-04-03

**Authors:** Behzad B. Pavri, Eitan Frankel

**Affiliations:** ^1^ Thomas Jefferson University Hospital Philadelphia Pennsylvania USA

**Keywords:** atrioventricular node ablation, atypical atrial flutter, supernormal conduction

## Abstract

We describe a patient who underwent AV node modification to create complete heart block in the setting of incessant, ablation‐and‐drug‐refractory, symptomatic atypical atrial flutter. His dual chamber defibrillator (previously implanted for resuscitated cardiac arrest) was programmed to the VVIR mode at a faster pacing rate of 85 bpm. Serendipitously, this rate was an almost exact factorial of his flutter rate of 250–260 bpm. This resulted in every 6th flutter wave falling in the supernormal period, resulting in fixed‐coupled supraventricular bigeminy and trigeminy in the setting of complete heart block. Reprogramming the pacing rate to 75 bpm abolished bigeminy and trigeminy.

## Graphical abstract

We describe supernormal conduction in a pattern of bigeminy or trigeminy due to serendipitous programming of the ventricular pacing rate at an exact factorial of the atrial flutter rate in a patient who underwent an ablate‐and‐pace strategy for recalcitrant atypical atrial flutter. Changing the ventricular pacing to a rate that was not an exact factorial of the flutter rate abolished this pattern.

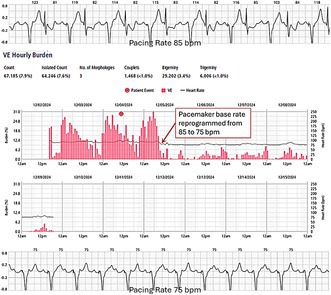

## 1 Case

**FIGURE 1:**
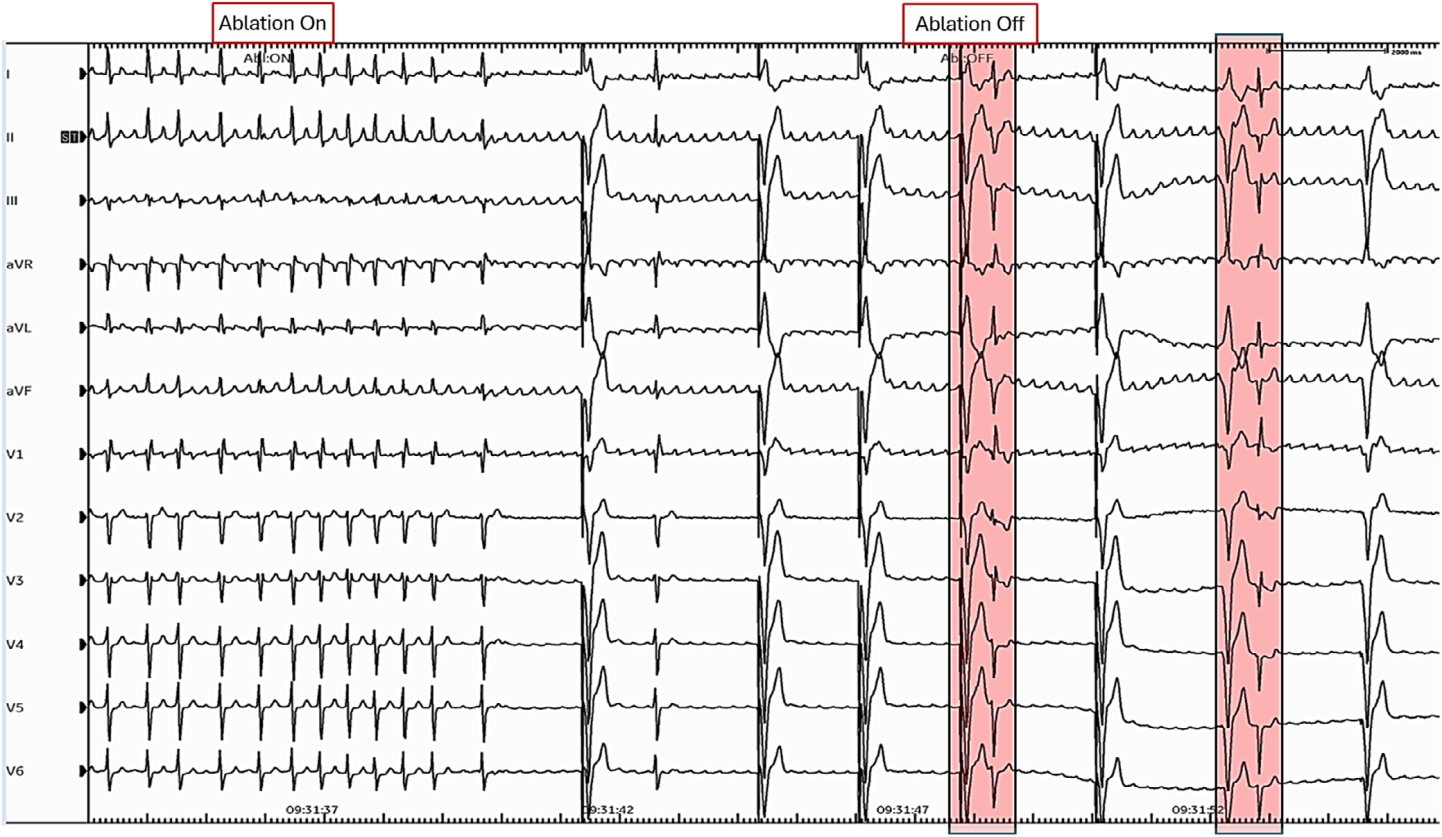
12‐lead ECG rhythm strip showing creating of complete heart block within 4 s of onset of radiofrequency ablation, with emergence of VVI pacing at 35 bpm. There are 2 narrow QUS complexes (shaded in pink) that show fixed coupling to the end of the T wave. See also Figure 6.

A 63‐year‐old man with hypertrophic cardiomyopathy and a primary prevention dual chamber defibrillator developed atrial fibrillation and atypical atrial flutter. Atrial flutter persisted in spite of multiple antiarrhythmic drug trials, pulmonary vein isolation, mitral isthmus ablation, cavotricuspid isthmus ablation (including inside the coronary sinus and vein of Marshall), roof lines, and finally surgical convergent procedure with atrial MAZE, left atrial excision, and epicardial cryoablation of the ligament of Marshall. Given his highly symptomatic, drug‐refractory atrial flutter, he underwent atrioventricular node modification (compact node ablation) with the creation of complete heart block. Occasional premature beats were noted immediately following heart block (Figure 1).

**FIGURE 2:**
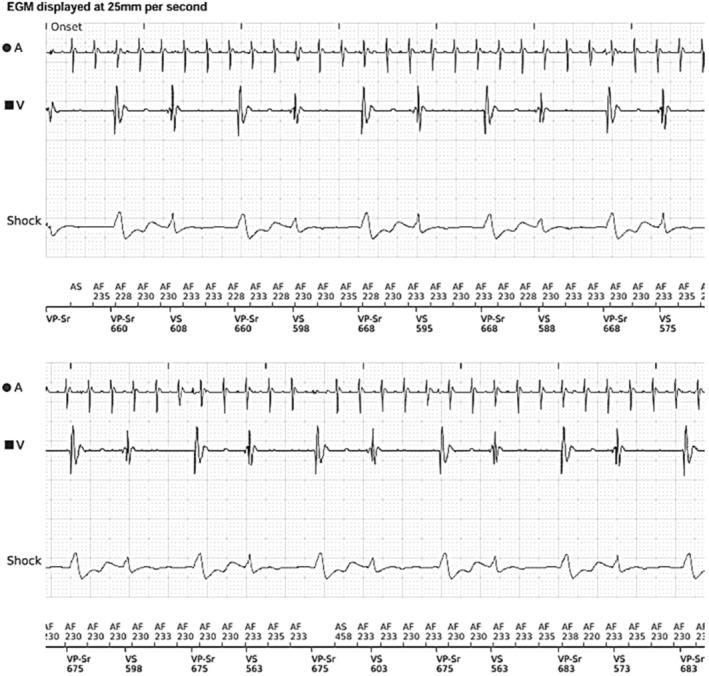
Section of remote transmission from dual chamber Boston Scientific defibrillator within 24 h of AV node ablation. There are ventricular pacing and native QRS complexes in a pattern of fixed coupled bigeminy at a coupling interval of about 580 ms. See also Figure 6.

Following the procedure, the patient was programmed to VVIR 85 bpm, and he continued to complain of palpitations and shortness of breath. His remote transmissions showed 58% ventricular pacing with periods of bigeminy, which were initially interpreted as *ventricular* bigeminy, as shown in Figure 2.

**FIGURE 3:**
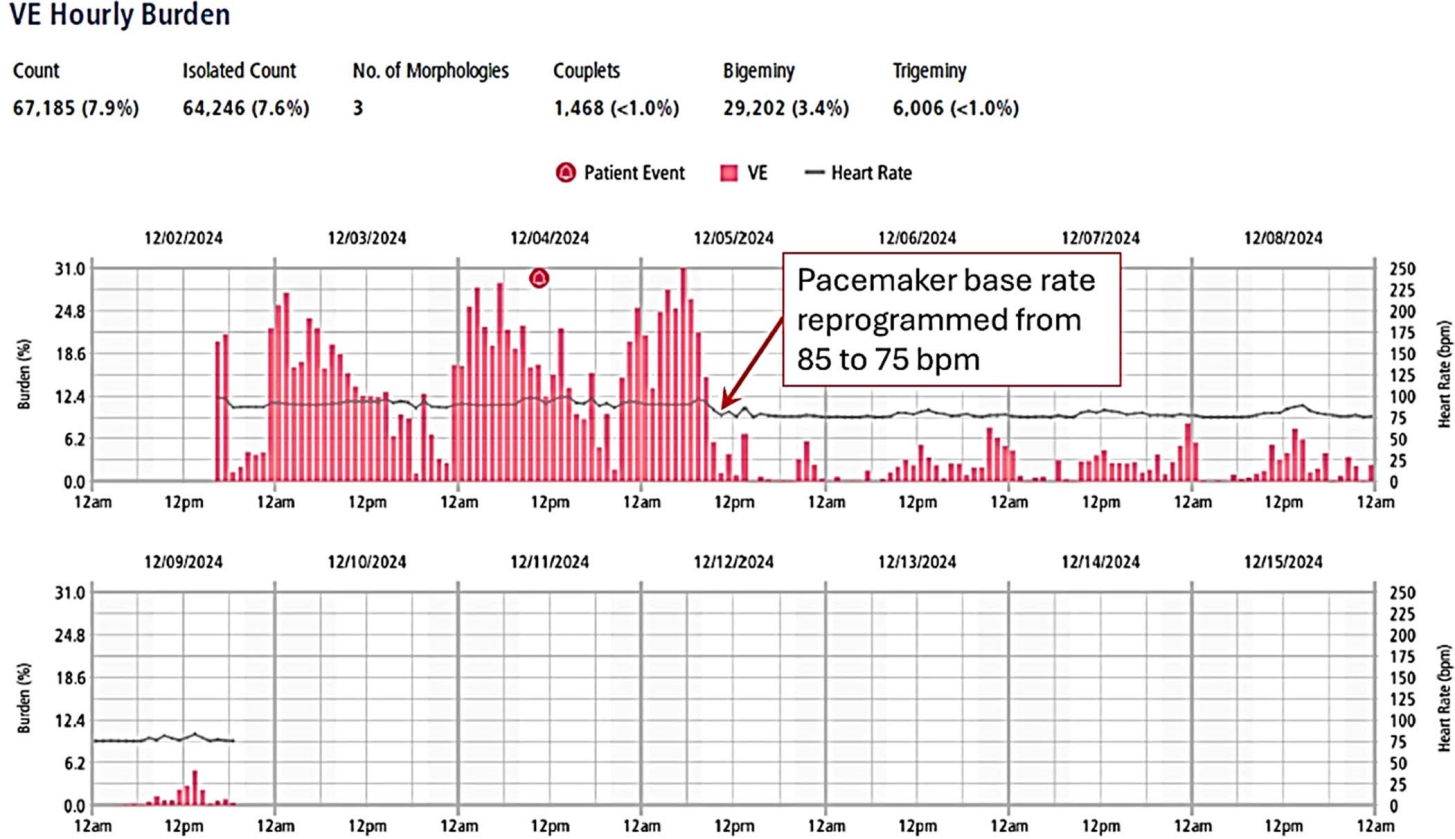
Heart rate trends from 7‐day Holter showing hourly burden of “ventricular” ectopy. Note the immediate reduction in ectopy burden coincident with the lowering of the base rate from 85 to 75 bpm, as marked by the arrow.

The patient was prescribed beta‐blockade, and when bigeminy persisted, sotalol, without improvement. An outpatient Holter was obtained for 1 week, but at day #2 of recording, *we suspected that the bigeminy was, in fact, supraventricular and possibly related to fixed‐coupled supernormal conduction of flutter impulses*, similar to what had been observed at the time of AV node ablation. He was brought in for defibrillator programming, and the base rate was lowered from 85 to 75 beats per minute. The patient reported improvement in symptoms. The Holter was completed, and the heart rate trends showed an immediate decline in the “ventricular” ectopy burden, as shown in Figure 3.

## 2 Analysis of Tracings

**FIGURE 4:**
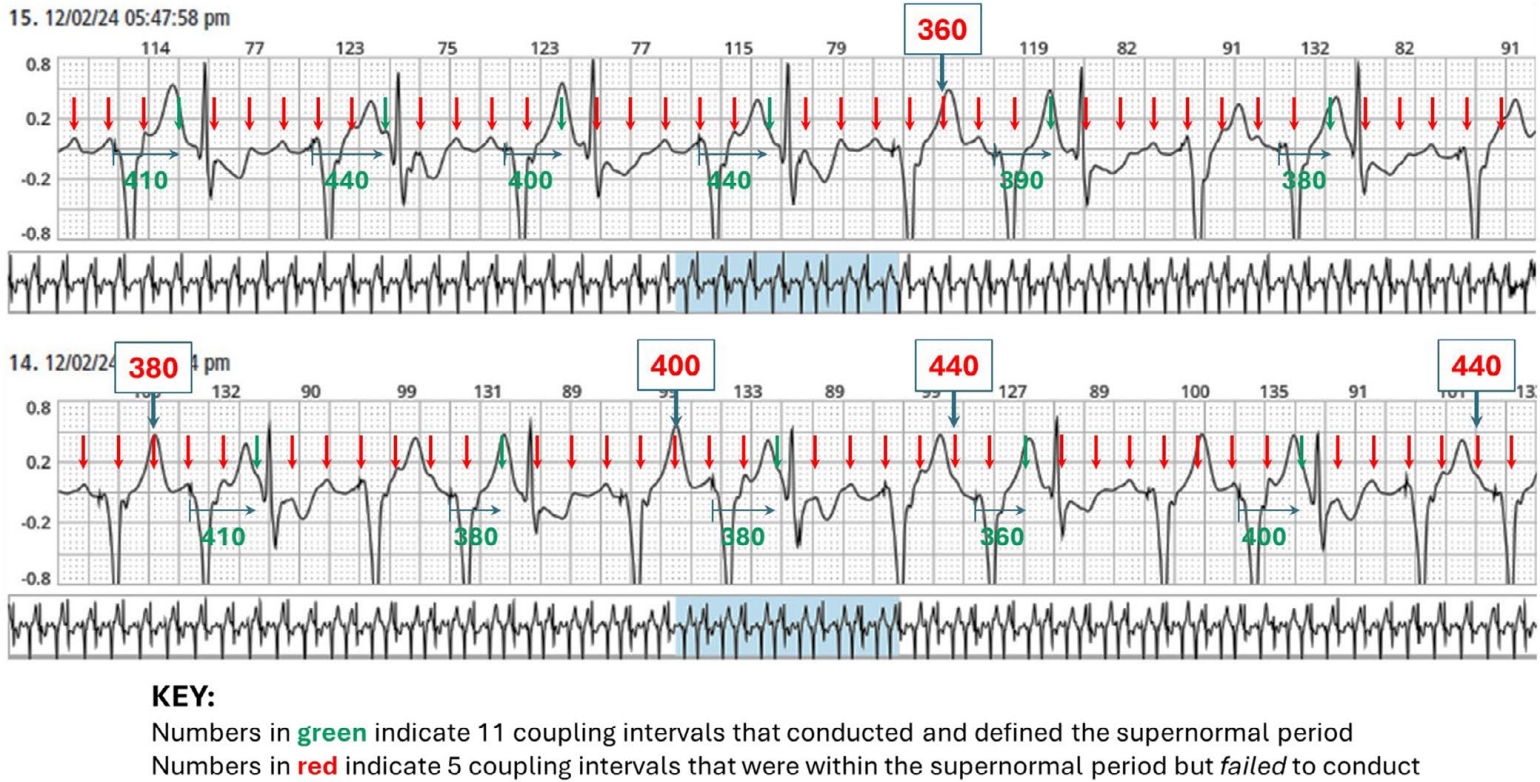
Two (non‐consecutive) Holter strips with flutter waves marked by red arrows. The top strip shows supraventricular beats in a pattern of bigeminy transitioning to trigeminy, whereas the bottom strip shows trigeminal coupling. There are 11 intervals (measured from the pacing spike to the flutter waves that are presumed to conduct within the window of supernormal conduction) shown in green. There are 5 intervals that fall within this window that do not conduct shown in red. All other flutter waves outside this window fail to conduct.

Sample ECG strips from the first 24 h of the Holter recording were analyzed. We measured 51 intervals from 2 (non‐continuous) Holter strips. Intervals were measured from each pacing spike to subsequent flutter waves, up to 650 ms later. The coupling intervals of the 11 flutter waves that resulted in supernormal conduction are shown in green numbers in Figure 4; this defined the window of supernormal conduction. The red numbers in Figure 4 represent 5 coupling intervals that were within the window of supernormal conduction but did not conduct.

With the flutter cycle length of 230–235 ms and the pacing rate set at 85 bpm (cycle length 706 ms), every 3rd flutter wave would be fixed coupled to the pacing spike (235 × 3 = 705). Because of ventricular refractoriness (QT interval of about 440 ms), on average, every 6th flutter wave fell in the supernormal window of conduction; rate‐responsive pacing slightly changed which flutter wave fell in the supernormal window.

When the base rate was decreased from 85 to 75 bpm (a cycle length of 800 ms), now every 14th flutter wave would be close to being a factorial of the pacing cycle length (800 × 4 = 3200; 230 × 14 = 3220), and would be “fixed coupled” to the paced QRS. Due to ventricular refractoriness, even fewer flutter waves fell within the window of supernormal conduction, and the number of premature supraventricular impulses decreased to < 3% for the remainder of the recording, as shown in Figure 3.

## 3 Discussion

**FIGURE 5:**
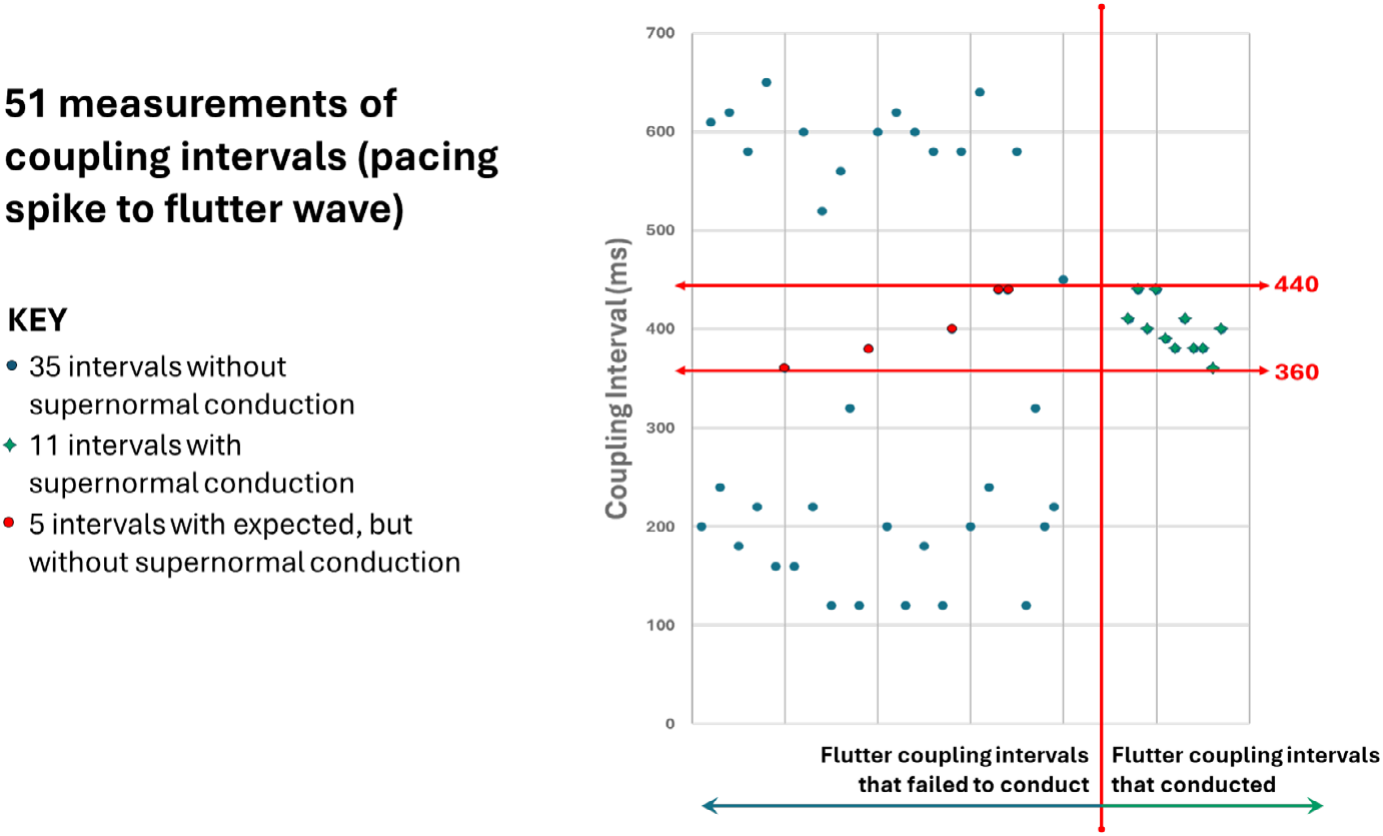
Graph of 51 intervals measured for 650 ms after each pacing spike from the 2 strips shown in Figure 4. The only coupling intervals that conduct to the ventricles are between 360 and 440 ms.

This is the first report of supernormality resulting in bigeminy and trigeminy due to the pacing rate being inadvertently programmed to a nearly exact factorial of the flutter rate. The supernormal conduction coupling interval “window” was mapped (by measuring 51 flutter waves that fell after 650 ms of the pacing spike). All 11 flutter impulses that conducted to the ventricles did so within a range of coupling intervals between 360 and 440 ms from the prior paced QRS complex, as measured from the pacing spike. However, there were 5 flutter impulses that fell within that same window of coupling that did *not* conduct to the ventricles, as shown in Figure 5. This intermittent failure of supernormal conduction may be attributed to the fickle nature of supernormality and the need for critically timed impulses that may vary based on ventricular repolarization.

The supernormal period is a brief period during electrical diastole when the transmembrane action potential is such that electrical conduction is more rapid than expected or occurs when block is anticipated (Moore et al. 1993). Thus, the term is in some sense a misnomer, in that conduction is not better than normal, but rather it is *better than expected*. The first description of supernormal conduction was by Lewis and Master in 1924 in a patient with complete heart block (Lewis and Master 1924).

When considering supernormal conduction as an explanation for the unexpected conduction of premature supraventricular impulses, one must always consider alternate explanations (Gallagher et al. 1973; Kenia et al. 2014); the differential diagnoses include:

1. *Gap phenomenon*, where proximal delay allows distal recovery (when shorter‐coupled atrial depolarizations conduct whereas later‐coupled atrial depolarizations do not, due to delay in the AV node allowing His‐Purkinje recovery)
2. *Infra‐nodal Wenckebach and equal delay* (when a bundle branch block pattern resolves with QRS narrowing due to equally slowed conduction in both bundle branches)
3. *Resolution of phase 4 block* (when bundle branch block at slow rates unexpectedly resolves with an atrial premature depolarization)
4. *Peeling back of refractoriness* (when 2:1 atrioventricular block may change to 1:1 atrioventricular conduction after a ventricular premature depolarization)
5. *Wedensky facilitation* is described in animal nerve preparations and possibly in humans (Fisch and Knoebel 1968) and relates to a strong impulse arriving at an area of block that lowers the excitation threshold and makes conduction possible. It may be invoked after long RR intervals (Friedberg 1971).

None of these alternate mechanisms are applicable to our case. Bigeminy in sinus rhythm with a malfunctioning pacemaker due to supernormality has been previously reported, where pacing outputs that failed to capture in diastole could capture only when they occurred in a narrow window of time at the end of the T wave during 2:1 AV conduction (Fisch 1990).

**FIGURE 6:**
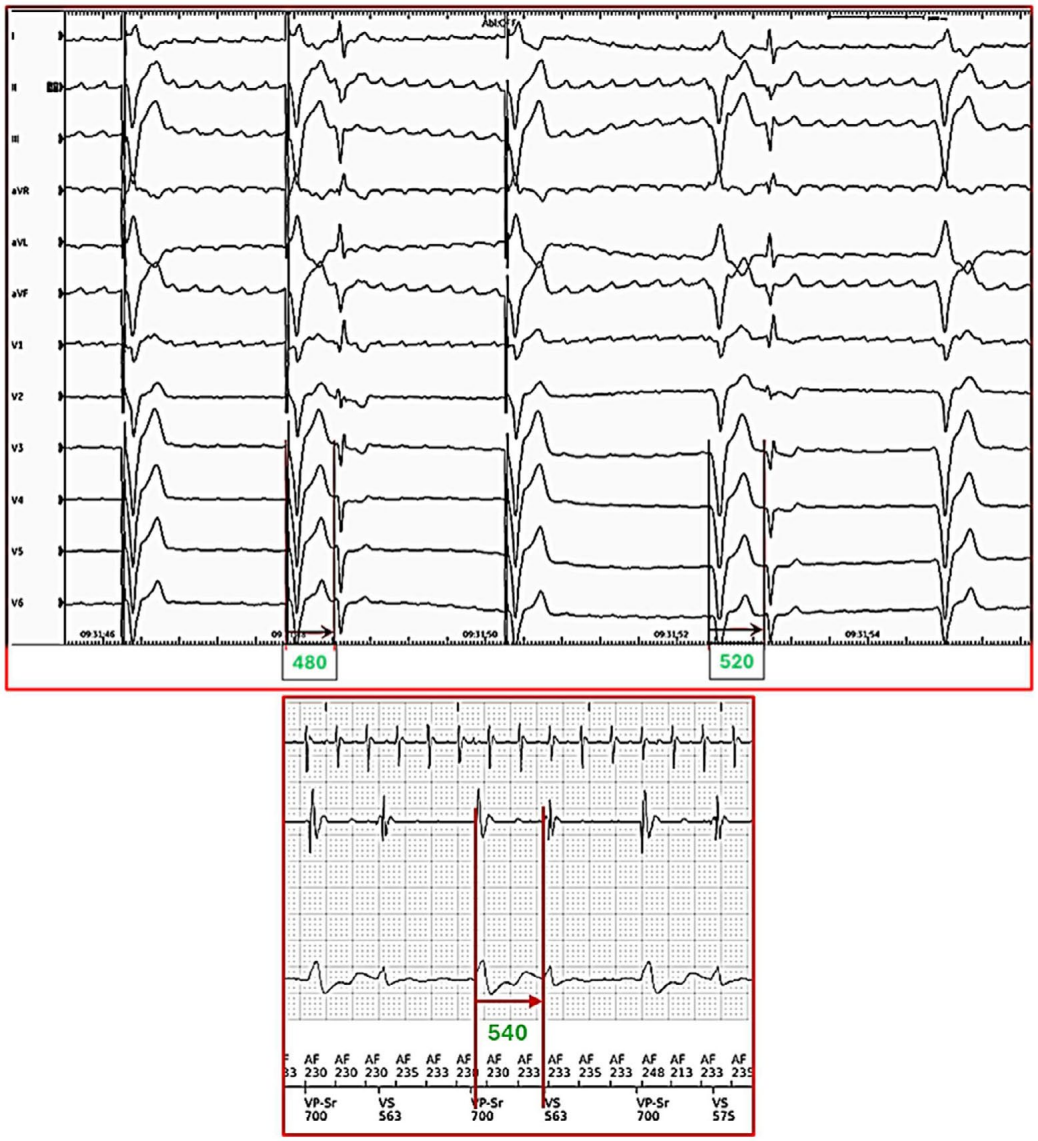
Enlarged section of Figures 1 and 2 to show measurements of coupling intervals that were conducted within the supernormal window of 480 to 540 ms. This coupling was longer than the window identified from the Holter because the QT interval was longer immediately following AV node ablation.

Additionally, in retrospect, we noted that supernormal conduction was occurring at a coupling interval of 480 to 540 ms immediately following the creation of complete heart block and as reported via remote transmission 24 h later, as shown in Figure 6.

At first glance, these coupling intervals were well outside the window of supernormal conduction that was seen on the Holter. However, this difference was related to the longer QT interval (about 480–520 ms) immediately following AV node ablation; as the QT interval shortened, so did the window of supernormal conduction.

Finally, premature ventricular contractions (PVCs) are unlikely to explain our observations given that the QRS complexes were relatively narrow, the patient had no PVCs prior to ablation (both in atrial flutter and in sinus rhythm), the coupling interval of the premature beats was linked to the ambient QT interval, and one would not expect the PVC burden to decrease coincidentally with the lowering of the pacing rate.

**In summary**, we describe supernormal conduction in a pattern of supraventricular bigeminy or trigeminy due to the serendipitous programming of the ventricular pacing rate at a nearly exact factorial of the atrial flutter rate. This resulted in very frequent supraventricular conduction after the ablation of the AV node and incorrect declaration of “ventricular bigeminy”, leading to inappropriate initiation of antiarrhythmic drugs. Once recognized, it was almost completely halted by changing the pacing rate to one that was not an exact factorial of the flutter rate. Supernormal conduction should be considered in the differential diagnosis for < 100% ventricular pacing and grouped ventricular depolarizations after atrioventricular node modification in patients with atrial flutter.

## Author Contributions

Dr. Behzad B. Pavri concieved, planned and wrote the 1st draft of the manuscript, and generated the figures. Dr. Eitan Frankel re‐wrote the manuscript, and helped with proof‐reading and data collection.

## Conflicts of Interest

The authors declare no conflicts of interest.

## Data Availability

The data that support the findings of this study are available from the corresponding author upon reasonable request.
